# Improvement of in vitro and early in utero porcine clone development after somatic donor cells are cultured under hypoxia

**DOI:** 10.1002/mrd.23132

**Published:** 2019-02-19

**Authors:** Bethany R. Mordhorst, Joshua A. Benne, Raissa F. Cecil, Kristin M. Whitworth, Melissa S. Samuel, Lee D. Spate, Clifton N. Murphy, Kevin D. Wells, Jonathan A. Green, Randall S. Prather

**Affiliations:** ^1^ Department of Animal Sciences University of Missouri Columbia Missouri

**Keywords:** blastocyst, cell culture, cloning, fetal development, hypoxia, litter size, metabolism, nuclear transfer, porcine/pig, pregnancy, reprogramming, somatic cell nuclear transfer

## Abstract

Genetically engineered pigs serve as excellent biomedical and agricultural models. To date, the most reliable way to generate genetically engineered pigs is via somatic cell nuclear transfer (SCNT), however, the efficiency of cloning in pigs is low (1–3%). Somatic cells such as fibroblasts frequently used in nuclear transfer utilize the tricarboxylic acid cycle and mitochondrial oxidative phosphorylation for efficient energy production. The metabolism of somatic cells contrasts with cells within the early embryo, which predominately use glycolysis. We hypothesized that fibroblast cells could become blastomere‐like if mitochondrial oxidative phosphorylation was inhibited by hypoxia and that this would result in improved in vitro embryonic development after SCNT. In a previous study, we demonstrated that fibroblasts cultured under hypoxic conditions had changes in gene expression consistent with increased glycolytic/gluconeogenic metabolism. The goal of this pilot study was to determine if subsequent in vitro embryo development is impacted by cloning porcine embryonic fibroblasts cultured in hypoxia. Here we demonstrate that in vitro measures such as early cleavage, blastocyst development, and blastocyst cell number are improved (4.4%, 5.5%, and 17.6 cells, respectively) when donor cells are cultured in hypoxia before nuclear transfer. Survival probability was increased in clones from hypoxic cultured donors compared to controls (8.5 vs. 4.0 ± 0.2). These results suggest that the clones from donor cells cultured in hypoxia are more developmentally competent and this may be due to improved nuclear reprogramming during somatic cell nuclear transfer.

## 1 INTRODUCTION

For two decades, since the creation of the cloned first lamb using a somatic cell donor, the scientific community has strived to improve the efficiency of somatic cell nuclear transfer (SCNT) in mammals (Campbell, McWhir, Ritchie, & Wilmut, 1996; Gábor, 2018). Notably, numerous live animals from 23 species have been successfully produced by SCNT due to an amalgamation of efforts from several dedicated researchers discovering new strategies (reviewed in Loi, Iuso, Czernik, & Ogura, 2016). Therefore efficiency has improved over time and breakthroughs are still occurring, where even the most challenging species are proving to be clonable (Liu et al., 2018). While other emerging techniques are improving, such as zygote injection of CRISPR/Cas9, SCNT is still one of the most efficient ways to reliably create genetically engineered animals. Despite the successful induction protocols for ESC and iPSC in rats (Liao et al., 2009) and mice (Bryja, Bonilla, & Arenas, 2006), creating reliable protocols has proven to be more challenging in the larger domestic species. A major hurdle for cow and pig iPSCs is that both 1) require continued expression of the introduced transgenes to maintain some degree of pluripotency, 2) have limited passage life, and 3) are not able to form teratomas in immunodeficient mice (reviewed in Ezashi, Yuan, & Roberts, 2016). Due to these challenges, SCNT is still popular in swine.

Gene‐edited and/or transgenic (GET) pigs serve as excellent models to study disease progression and develop treatments for human genetic disorders. The pig, in particular, conveys great similarity to humans in their anatomy, physiology, and genomics, therefore, allowing them to exhibit symptoms of human pathologies more accurately and reliably than other animal models (reviewed in Fan & Lai, 2013; Prather, Lorson, Ross, Whyte, & Walters, 2013; Walters et al., 2012). For several reasons, GET pigs are the most likely donor option for future xenotransplantation of organs; therefore great efforts have been underway to make this possibility a reality (Bottino et al., 2014; Ekser, Rigotti, Gridelli, & Cooper, 2009; Klymiuk, Aigner, Brem, & Wolf, 2010; Lai et al., 2002; Lavitrano et al., 2002; Lutz et al., 2013; Mohiuddin et al., 2014; L. Yang et al., 2015). Due to the usefulness of GET pigs in both biomedicine and agriculture, there is a growing need for the creation of new or improved upon models, which at this time, is still predominately achieved via SCNT. Currently, the efficiency of SCNT in pigs is approximately 1–3% (Whitworth & Prather, 2010). This percentage varies slightly depending on if investigators calculate success rate based on total embryos reconstructed, total embryos transferred, or pregnancy rate of surrogates to which embryos were transferred. Nevertheless, this rate is low and there is a substantial need for improvement. When blastomeres are used as donor cells for SCNT the rate of success is significantly improved (Mitalipov, Yeoman, Nusser, & Wolf, 2002). Therefore we speculated that if somatic cells could be induced to be more blastomere‐like, the cloning efficiency may be greatly improved.

There is growing evidence that cellular reprogramming is facilitated in part by upregulation of glycolysis (Folmes et al., 2011; Kondoh et al., 2005; Moussaieff et al., 2015; Zhu et al., 2010). In light of this, we reasoned promoting a highly glycolytic metabolism could facilitate nuclear reprogramming. Somatic cells predominately use mitochondrial oxidative phosphorylation and the citric acid cycle for the production of energy whereas the metabolism of preimplantation embryos is evidenced to be more glycolytic and Warburg effect‐like in nature (Krisher & Prather, 2012). We hypothesized that if donor fibroblasts were cultured under hypoxic conditions, it would elicit the higher glycolytic activity thereby promoting metabolism exhibited in the blastomeres of early embryos. We speculated this would aid in the facilitation of nuclear reprogramming and improve cloning efficiency. In this study, we investigated if restricting oxygen from donor fibroblasts during cell culture would improve measures of in vitro developmental quality in reconstructed pig embryos and improve survival through early gestation.

## 2 RESULTS

Fusion of reconstructed embryos was not significantly different (*p* = 0.10) between hypoxic (HYP) and control (CON) cultured fibroblast donors and was not different amongst cell lines (*p* = 0.44). While the total proportion of embryos cleaved was not different between donor fibroblast oxygen culture treatments (*p* = 0.75), HYP reconstructed clones had a higher (*p* = 0.03) proportion cleave earlier (within the first 24 hr) compared to control in both experiments (Table 1). Likewise, a higher proportion of CON embryos cleaved later (at least by 44 hr) than HYP (*p* = 0.04; Table 1). Total, early, and late cleavage was not significantly different amongst cell lines which were used in embryo transfer experiments (*p* ≥ 0.06) however some of these measures were lower in the cell line used for preliminary in vitro experiments (*p* < 0.01).

**Table 1:** In vitro development and gestational Day 35 survival of clones from donor cells cultured in hypoxia or as controls

	Treatment^a^				
	Hypoxia	Control	Standard error	*p*‐Value	
Fusion^b^ (%)	85.2	82.8	1.0	0.10	
Early cleavage^c^ (%)	61.6	57.2	1.4	0.03*	
Later cleavage^d^ (%)	15.5	18.6	1.1	0.04*	
Total cleavage^e^ (%)	77.1	76.1	1.1	0.53	
Total blastocyst development^f^ (%)	36.4	30.9	1.3	<0.01*	
Blastocyst cell number^g^	52.7	35.1	3.0	<0.0001*	
Survival probability^h^ (%)	8.5	4.0	0.2	0.03*	

^a^ In vitro development measures of somatic cell nuclear transfer (SCNT) embryos from donor cells cultured in hypoxia (hypoxic; 2 days at 5% oxygen, 1 day at 2.5% oxygen, 4 days at 1.25% oxygen) or as controls (control; 5% oxygen 7 days).

^b^ Percentage of embryos successfully fused from total reconstructed embryos of SCNT embryos.

^c^ Percentage of embryos successfully cleaved within 24 hr from SCNT.

^d^ Percentage of embryos successfully cleaved within 44 hr from SCNT excluding those formed by 24 hr.

^e^ Total percentage of embryos successfully cleaved after SCNT.

^f^ Percentage of embryos successfully forming blastocyst stage embryos within 7 days from SCNT.

^g^ Number of cells in blastocyst stage SCNT embryos.

^h^ Number of favorable outcomes (viable fetuses) from total events (all embryos transferred) expressed as a percentage (multiplied by 100).

**Figure 1:**
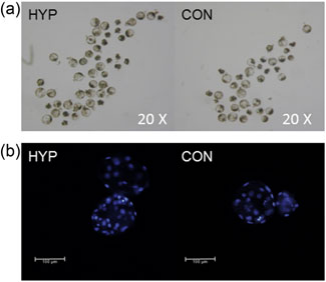
Representative images of developmental Day 7 somatic cell nuclear transfer embryos from donor cells cultured in hypoxia (HYP; 2 days at 5% oxygen, 1 day at 2.5% oxygen, 4 days at 1.2% oxygen) or as controls (CON; 5% oxygen 7 days). (a) 4× magnification of SCNT embryo groups separated out after having cleaved early inside 4‐well plates during culture derived from hypoxic and control cultured donor cells. (b) 20× magnification of single SCNT blastocysts stained with Hoechst 33342 derived from hypoxic and control cultured donor cells in the process of hatching

**Figure 2:**
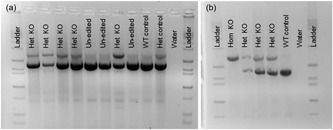
Examples of Day 35 fetal genotyping using PCR amplification to detect editing of sialoadhesin (*SIGLEC1*/ *SN*/ *CD169*). Donor cell lines used in early gestation survival experiments were homozygous or heterozygous *SIGLEC1* knockouts or had no editing (wild type). (a) Example of litter from heterozygous knockout and wild type cell lines used in somatic cell nuclear transfer. Corresponds to the litter in the last row of Table 2. (b) Example of litter from heterozygous and homozygous knockout cell lines used in somatic cell nuclear transfer. Corresponds to the litter in the first row of Table 2. PCR: polymerase chain reaction

Blastocyst production was improved when embryos were created from HYP donors (*p* < 0.01; Table 1 and Figure 1). Furthermore, blastocysts from HYP donors contained more nuclei than CON (*p* < 0.0001; See Table 1 and Figure 1). Blastocyst production was lower in the homozygous knockout (KO) cell line (*p* ≤ 0.01; 24%) and similar amongst the other cell lines used (*p* ≥ 0.15; average 33%; SE = 2%). In utero, the homozygous KO line had lower survival than the heterozygous KO line (*p* = 0.01; 0.3 ± 2.9 vs. 9.4 ± 1.6%) but not different from the Ossabaw line (*p* = 0.49; 4.27; Figure 2 provides examples of Day 35 fetal genotyping for sialoadhesin [*SIGLEC1*] editing). Ossabaw and the heterozygous KO line did not have significantly different survival in utero (*p* = 0.05). From 446 embryos transferred into 12 surrogates, 6 surrogates became pregnant carrying 20 HYP and 9 CON fetuses (Table 2). Fetuses collected were similarly sized amongst treatments (*p* = 0.17; CON = 3.16 ± 0.14 cm; HYP = 2.93 ± 0.09 cm) and cell lines (*p* = 0.06; OSS = 3.30 ± 0.16 cm; heterozygous KO = 2.90 ± 0.08 cm; homozygous KO = 3.5 ± 0.39 cm). Survival probability was increased when clones were from HYP versus CON donors (*p* = 0.025; Table 1). This corresponded to a 2.5‐fold increase in overall system efficiency (blastocyst development rate * fetal survival rate) in HYP clones versus CON (3.0% vs. 1.2% efficiency).

**Table 2:** Gestational Day 35 fetal retrievals from somatic cell nuclear transfer of hypoxia and control culture treated fibroblasts

Gilt recipient cycle day	Number of embryos transferred	Developmental day of blastocysts	Total litter size	Number of control^a^ fetuses	Number of hypoxia^b^ fetuses
4	40	5	4	3	1
3	32	5	6^c^	0	6
5	36	6	5^d^	1	4
4	40	6	8	2	6
4	40	6	3	0	3
4	36	6	3^d^	3	0

^a^ Fetal fibroblasts used in somatic cell nuclear transfer (SCNT) were cultured in 5% oxygen for 7 days.

^b^ Fetal fibroblasts used in SCNT were cultured in hypoxia: 2 days at 5% oxygen, 1 day at 2.5% oxygen, 4 days at 1.25% oxygen.

^c^ Uterus also contained two regressed fetuses: one hypoxia and one control.

^d^ Uterus also contained two regressed fetuses: three hypoxia and two control.

## 3 DISCUSSION

In an effort to improve cloning, several studies have attempted to elucidate what constitutes the ‘ideal donor cell.’ These studies include investigations of serum starvation and cell cycle regulation, type of cell, age of animal from which cells were extracted, age of cells themselves (or passage number), epigenetic reprogramming, degree of pluripotency, and antioxidant treatment of cells (Bonk et al., 2007; Campbell et al., 1996; Chen et al., 2015; Dominko et al., 1999; Heyman et al., 2002; Iager et al., 2008; Kato, Tani, & Tsunoda, 2000; Mitalipov et al., 2002; Oback & Wells, 2007; Powell et al., 2004; Tani, Kato, & Tsunoda, 2001; Wakayama & Yanagimachi, 2001; Wells et al., 2003; Whitworth, Zhao, Spate, Li, & Prather, 2011; Wilmut et al., 2002; X. Yang et al., 2012). Proper nuclear remodeling and epigenetic reprogramming of donor cells is revered to be the key to improving SCNT and is thoroughly under examination (reviewed in Armstrong, Lako, Dean, & Stojkovic, 2006; Niemann, Tian, King, & Lee, 2008; Whitworth & Prather, 2010). Improper nuclear reprogramming has also been attributed to the developmental defects which are sometimes observed in clones; the genome of somatic cells must be reset to express genes consistent with appropriate progression of embryonic development (reviewed in Armstrong et al., 2006). In accordance with this, when blastomeres are used as donor cells for SCNT the rate of success was improved (Mitalipov et al., 2002).

We hypothesized that if porcine fetal fibroblasts (the most common cell type used for cloning of pigs) were cultured under hypoxic conditions the metabolism of the donor cells would become more blastomere‐like and thus it might improve reprogramming and subsequent cloning efficiency. Therefore we designed these experiments to test our hypothesis and see if this mechanism (hypoxic culture) would be effective and worth pursuing further. It was desirable that this ‘reprogramming’ induction be through a simple mechanism that could be applied to any cell line, such as altering the metabolism during culture via hypoxia. Previously our laboratory reported that oxygen restriction in porcine fetal fibroblasts (the same line used in in vitro experiments in the current study) induced expression of *hexokinases 1* and *2*, *glucose‐6‐phosphate isomerase*, *glyceraldehyde‐3‐phosphate dehydrogenase*, *triose phosphate isomerase 1, aldolase, fructose‐bisphosphate C*, and *phosphoglycerate kinase*; all enzymes involved in glycolysis (Mordhorst, Murphy, Ross, et al., 2018). In addition, expression of *pyruvate dehydrogenase kinase 1* was also induced, this kinase is evidenced to be a key player in thwarting the entrance of pyruvate into the TCA cycle thereby effectively decreasing mitochondrial oxidative phosphorylation (Papandreou, Cairns, Fontana, Lim, & Denko, 2006). Hypoxia treatment results in metabolism being shifted to become more blastomere‐like (Mordhorst, Murphy, Ross, et al., 2018; Redel et al., 2012).

In this study, we demonstrate that measures of in vitro development were improved when fibroblasts cultured in hypoxia were used as donors for SCNT. Clones from HYP fibroblasts consistently cleaved earlier and produced more blastocysts with higher cell numbers (Table 1; Figures 1, 2) regardless of the fibroblast line used for cloning. Early cleavage is a predictor of developmental competence and embryo quality (Cruz et al., 2012; Isom, Whitworth, & Prather, 2012; Kaith et al., 2015). Additionally, early cleaving embryos have better rates of pregnancy (Lundin, Bergh, & Hardarson, 2001).

The improvement in blastocyst production from HYP donors is modest (5.5%) however this translated to a 1.5‐fold increase in cell number and 2.1‐fold increase in the probability of survival (fetuses attained). Overall these changes lead to a 2.5‐fold increase in system efficiency (blastocyst development rate * fetal survival rate). In this study, our Day 35 pregnancy rate was 50% (6 of 12 surrogate gilts became pregnant) which is higher than previous experiments in our laboratory involving the same personnel (41.6% and 28.6%; Mordhorst, Murphy, Schauflinger, et al., 2018) through the average number of fetuses per litter was higher compared to the current study (5.9 vs. 4.8 fetuses/litter). One limitation of this study is that at the present time, no piglets have been delivered to term using hypoxic donors. We did choose to retrieve fetuses at 35 days of gestation based on research from others indicating that 30% of conceptus loss occurs in gestation Days 10–30 in pigs (Wessels, Linton, Croy, & Tayade, 2007).

There were cloning differences between fibroblasts derived from the same fetal cell line based on whether they were heterozygous or homozygous Sialoadhesin knockouts (*SIGLEC1* KO). The homozygous *SIGLEC1* KO line produced few Day 5 blastocysts, limiting the number of embryos we could equally transfer from each treatment. Of the two replicates, two of the four gilts became pregnant and whereas they were not transferred on the same day, between the two gilts all combinations of the homozygous and heterozygous KO lines and donor treatments were represented. Due to the decreased blastocyst production however we decided to use a different cell line (Ossabaw) in combination with the *SIGLEC1* heterozygous KO line, a line proven to be ‘clonable’ and yield live animals which has been used for editing genes by the National Swine Research and Resource Center (RRID NSRRC:0008; https://nsrrc.missouri.edu/). According to another study from Kuhholzer, Hawley, Lai, Kolber‐Simonds, and Prather, (2001) clonal transgenic lines derived from the same pig fetus had different efficiencies of fusion, but also developed to the morula and blastocyst stages. One possibility is complications during embryonic development due to the gene‐altered; however, in our experience, these *SIGLEC1* KO pigs have normal gestational survival that would indicate the gene modification would not be a hindrance.

As this study was exploratory, additional studies in the field are needed. Our results indicate that using HYP donors improves cloning efficiency across different fetal fibroblast cell lines. Given the promising early results within our laboratory, research is warranted to investigate whether these results are replicable in other laboratories for other porcine cell line sources as well as in other species to determine if nuclear reprogramming is occurring. Further exploration is necessary to pinpoint effective duration of hypoxic culture and whether stepwise decreases in oxygen concentration are necessary. In this study, we used a hypoxic culture method which we knew to be effective in inducing a hypoxic cellular phenotype and changes in gene expression (Mordhorst, Murphy, Ross, et al., 2018).

In the present study, we did not note any differences in blastocyst development or Day 35 pregnancy rates between the use of hypoxic atmosphere incubators or hypoxic chambers. We switched to hypoxic chambers due to the feasibility of use as well as cost. One limitation is that we did not have a system to monitor how much oxygen was in chambers after gassing, whereas we could monitor oxygen in real time with the incubator system. It is possible using the hypoxia chambers that oxygen could go below 1.2%, but using that apparatus we could not regularly monitor oxygen content with our current instrument. However, the consumption of oxygen by two T25 flasks would presumably not be enough to notably further deplete the chamber's oxygen content.

Elucidation of molecular mechanisms as to how hypoxic culture is able to enhance SCNT would offer insight to cellular metabolic reprogramming and could shed light on other means to be pursued in achieving improved cloning efficiencies in the future. To our knowledge, this is one of the first times cloning efficiency of pigs has reached this rate aside from a study by Walker et al., where efficiency was reported as 5.5% (28 piglets/511 SCNT embryos transferred). In this study, in similar terms, of the 223 embryos from HYP donors, there were 20 fetuses (~9%); the caveat being future studies must be conducted which carry HYP clones to term to ascertain this rate of efficiency holds true. However, at the present, we thought that these results were novel and worth reporting to the scientific community to open further exploration into mechanisms and effectiveness of hypoxic culture on donor cells. From these experiments, we propose that the clones from donor cells cultured in hypoxia may be developmentally competent and this may be due to improved nuclear reprogramming during somatic cell nuclear transfer. Implications of our results may be a method for improved efficiency for cloning of pigs.

## 4 MATERIALS AND METHODS

Chemicals and materials were purchased from Sigma‐Aldrich, St. Louis, MO unless otherwise specified.

### 4.1 Animal care and compliance with ethical standards

All procedures performed in studies involving animals were approved and conducted in accordance with the ethical standards of the University of Missouri Institutional Animal Care and Use Committee at the University of Missouri in Columbia, Missouri. This study does not contain any experiments with human participants.

### 4.2 Fetal‐derived fibroblast cell culture

Porcine fetal fibroblast cell lines collected from dorsal regions (fetal shoulders to the rump/tail head) used in the study were established from d 35 pregnancies and utilized in previous experiments in our laboratory (Mordhorst, Murphy, Schauflinger, et al., 2018; Prather, Rowland et al., 2013). A cryogenic vial of fibroblasts (0.5 ml aliquots; ~1.5 million/ml in media containing 90% fetal bovine serum (FBS) and 10% dimethyl sulfoxide) was defrosted from liquid nitrogen storage for every replicate in the experiment. Cells were thawed and cultured in Dulbecco's modified Eagle's medium (1 g/L glucose with phenol red) supplemented with 15% FBS (Corning, Corning, NY) for seven days in T25 flasks (Corning). Cells were either treated as controls (CON) cultured in 5% oxygen for the duration of 7 days or cultured in step‐wise decreasing concentrations of oxygen (HYP) where for 2 days they were maintained at 5% oxygen, on the third day cultured at 2.5% oxygen, and from the fourth to the seventh days cultured at 1.2% oxygen. Hypoxic culture conditions were achieved one of two ways; either via increased nitrogen gas concentration in a standard mixed gas incubator or by using purchased preanalyzed mixed gas cylinders to fill humidified modular incubator chambers, which were placed in incubators.

When whole incubators were used (three initial in vitro experiment replicates and two replicates of embryo transfer experiments), a system of four nitrogen tanks connected by one gas line was assembled to ensure adequate nitrogen gas was available to incubators. The regulators of nitrogen tanks were set to sequentially empty the tanks one by one as culture experiments required a large quantity of nitrogen gas, and at least one of the tanks had to be exchanged for a full tank daily. During this, oxygen concentration of incubators was regularly monitored by a handheld gas monitor (model: InControl 1050; Labotect Labor‐Technik‐Göttingen GmbH; Göttingen, Germany) which was professionally tested by Utility Lab Services (part of Thermo‐Fisher Scientific; Waltham, MA) and proved to have an error of ±0.05% oxygen. Therefore the low oxygen treatment may have been as high as 1.25% or as low as 1.15% oxygen. Incubators were maintained at 38.5°C with a humidified atmosphere of 5.5% carbon dioxide during experiments.

In experiments where the modular incubator chamber (model MIC‐101; Billups‐Rothernberg, Inc.; Del Mar, CA) was used (four replicates of embryo transfer experiments), petri dishes of sterile water were placed in the bottom of the chamber below the grate to provide humidity. Vented cell culture flasks were placed inside chambers, which were then sealed and flooded with mixed gas for several minutes to displace gas within and achieve hypoxic conditions. Afterward, air ports were sealed and chambers were placed in the standard incubators with controls.

Cells from all treatments were plated at the same initial densities between treatments and either passaged on Day 5 of growth and plated at the same density (if needed) or had gas‐equilibrated media changed and allowed to grow 7 days consecutively. Passaging was done quickly, and dissociation reagents were kept cold in an effort to decrease the metabolism of cells and prevent the metabolism of oxygen as much as possible. Media that cells were plated in had been equilibrated to desired oxygen concentrations by incubating media in the incubators in petri dishes or within modular incubator chambers for an hour or more. At passaging, flasks were briefly rinsed with phosphate buffered saline (PBS) + 0.01 M ethylenediaminetetraacetic acid, and fibroblast cells were dissociated from flasks by brief incubation (37°C) with 1× TrypLE Express (Gibco, Invitrogen purchased from Thermo Fisher Scientific, Waltham, MA). Fibroblasts were pelleted (5 min at 500*g*) and either diluted and replated (Day 5) or approximately 2,000 cells/treatment were collected and placed in droplets to be used for selection during SCNT (Day 7).

### 4.3 Somatic cell nuclear transfer and embryo culture

Methods for SCNT were previously reported by (Mordhorst, Murphy, Schauflinger, et al., 2018). Sow‐derived oocytes were purchased from DeSoto Biosciences (Seymour, TN) and shipped overnight in maturation medium (90% M199 and 10% follicular fluid supplemented with 0.57 mM cysteine, 5 μg/ml insulin, 10 ng/ml epidermal growth factor, 5 μg/ml LH and FSH). After maturation (40–42 hr), cumulus cells were removed by gentle vortexing in the presence of 0.1% hyaluronidase. The average percentage of oocyte maturation across replicates in experiments was 72.9% (standard deviation of 3.1%). Oocytes were selected for enucleation based on the presence of a polar body and uniform cytoplasm. Oocytes were placed in a manipulation medium (Lai & Prather, 2003) supplemented with 7.0 µg/ml cytochalasin B during oocyte manipulation. In all experiments, SCNT was performed by two technicians and each treatment was split evenly amongst the technicians. Order of treatment in which SCNT was performed first was selected randomly. A hand‐tooled thin glass capillary was used to remove the polar body along with a portion of the adjacent cytoplasm (presumably containing the metaphase II plate) and a donor cell was placed in the perivitelline space.

Afterward, reconstructed embryos were fused in fusion medium (0.3 M mannitol, 0.1 mM CaCl_2_, 0.1 mM MgCl_2_, 0.5 mM HEPES buffer, pH 7.2) by two DC pulses (1‐s interval) at 1.2 kV/cm for 30 µsec using a BTX Electro Cell Manipulator (Harvard Apparatus, Holliston, MA). After electric pulse fusion, fused embryos were also chemically activated with 200 µM thimerosal (in 1× PVA‐TL Hepes) for 10 min in the dark and 8 mM dithiothreitol (in 1× PVA‐TL Hepes) for 30 min (Machaty, Wang, Day, & Prather, 1997). Embryos were then incubated in MU1 (Redel, Tessanne, Spate, Murphy, & Prather, 2015) with a histone deacetylase inhibitor 0.5 µM Scriptaid, for 14–16 hr in a 5% carbon dioxide (atmospheric oxygen) incubator (Whitworth, Zhao, Spate, Li, & Prather, 2011; Zhao et al., 2009). The next day, the SCNT embryos were moved into new MU1 culture media (without scriptaid) and placed in a low (5%) oxygen incubator with 5% carbon dioxide. During culture, embryos were inspected for the timing of cleavage (early: 24 hrs postactivation; late: at least by 44 hr) and blastocyst formation. Blastocyst stage SCNT embryos were fixed with 4% paraformaldehyde (16% paraformaldehyde diluted to 4% using 1× PVA‐TL Hepes) and stained with Hoechst 33342 on Day 7 of development for quantification of cell number in the first experiment (three replicates).

In vitro development data were compiled from two experiments: Experiment 1 consisted of vitro development only; in Experiment 2 in vitro development data was collected from the embryos transferred to surrogates. Therefore between the experiments, there were nine biological replicates and 2,525 oocytes used in SCNT to investigate whether clones from oxygen restricted donor cells had improved in vitro development. In both experiments gestational Day 35 fetal fibroblasts were used, however different cell lines were used. In Experiment 1 a single cell line was used in SCNT (BRM3P1; Mordhorst, Murphy, Ross, et al., 2018). In embryo transfer experiments, cell lines proven to produce live offspring were used to distinguish (via genotyping; Figure 2) which donor cell treatments survived in utero. Heterozygous or homozygous deletions of sialoadhesin (*SIGLEC1*/*SN*/*CD169*; Prather, Rowland, et al., 2013) or WT fibroblasts (a proven Ossabaw fetal fibroblast line, RRID NSRRC:0008) were cultured under both oxygen treatments and used as donor fibroblasts for SCNT. The homozygous cell line did not produce blastocysts well, which limited the number of embryos that could be transferred equally to surrogates. Due to this, we began using the WT fibroblast line because previous use had evidenced that it produced blastocysts well for various edits by other researchers.

### 4.4 Embryo transfer

Blastocyst‐stage embryos (*N* = 16–20 per SCNT fibroblast donor culture treatment) were surgically transferred into the oviductal ampullary‐isthmic junction of surrogate gilt recipients (3–5 days postobserved estrus). Surgical embryo transfer technique was conducted as previously established within our laboratory (Lai & Prather, 2003; Mordhorst, Murphy, Schauflinger, et al., 2018). For embryo transfer experiments, two surrogate gilts were used in each replicate (six replicates; 12 gilts) in a complete‐block type fashion where between the gilts each cell line (two of the three different lines utilized) and donor cell culture treatment (hypoxia and control) were used. Two ‘sets’ of gilts became pregnant as well as two single gilts from other replicate sets became pregnant.

### 4.5 Fetal collection and genotyping

Surrogates were humanely euthanized via ear vein infusion of Euthasol (Virbac AH, Inc; Fort Worth, TX) at 35 of embryonic development. The reproductive tract was excised from the cervix to ovaries and opened along the entire length of uterine horns for fetal collection. Tissue was excised from fetuses and washed in 1× PBS then frozen until DNA extraction and genotyping. To establish which donor cell treatment survived better in utero, fetuses were genotyped (Figure 2) via polymerase chain reaction amplification for the presence of *SIGLEC1* alleles using the same primers and procedure used in Prather, Rowland et al., (2013). When bands were not clearly obvious, DNA was extracted from frozen samples again and sent for sequencing verification at the University of Missouri core facility.

### 4.6 Statistical analysis

For in vitro developmental measures, nine replicates (three in vitro cultured only and six used for embryo transfer; 2,525 total oocytes) were used in analyses. Three replicates of the same donor cell line were used to compare cell number within blastocysts for donor cell oxygen culture treatments (112 total blastocysts). Embryo transfer data consisted of 446 total blastocysts transferred into 12 surrogates (6 replicates of surrogate pairs). Data distributions and Shapiro–Wilk test of normality values were generated using the Univariate procedure of SAS (SAS, Cary, NC); log or square root transformations were made before statistical analysis where necessary to meet assumptions for analyses. All data were analyzed using a MIXED procedure in SAS for main effects of donor cell treatment and cell line; interactions were assessed however this effect was removed from analyses when found to be insignificant. For embryo transfer data, blastocysts transferred were nested within surrogate gilt and a random effect of replicate was used (mainly because it corresponds to the time/season differences for this study duration). Survival probability was considered the number of favorable outcomes (viable fetuses) from the total (embryos transferred). Analyzed variables were considered statistically different if the *p* < 0.05.

## CONFLICT OF INTERESTS

The authors declare that there are no conflict of interests.
